# Toxic Anterior Segment Syndrome After an Uncomplicated Vitrectomy With Epiretinal Membrane Peeling

**DOI:** 10.7759/cureus.14464

**Published:** 2021-04-13

**Authors:** Piotr Kanclerz

**Affiliations:** 1 Ophthalmology, Hygeia Clinic, Gdansk, POL

**Keywords:** endophthalmitis, epiretinal membrane, toxic anterior segment syndrome, pars planitis, vitrectomy

## Abstract

Infectious endophthalmitis is the most devastating complication of eye surgery and is associated with severe inflammation of ocular tissues. This study aimed to present a similar condition, a case of toxic anterior segment syndrome (TASS) after an uncomplicated vitrectomy.

A 69-year-old woman presented with epiretinal membrane and underwent 25-gauge pars plana vitrectomy with membrane peeling in her left eye. Thirty hours after the procedure, the patient complained of increasing loss of visual acuity and a red left eye. The ophthalmic examination revealed moderate hyperemia, hypopyon and snowbanks in the anterior vitreous. Subconjunctival and topical steroids were administered, and the inflammatory symptoms resolved within 30 days. The visual acuity improved to 20/32, however, cystoid changes were noted in the macula by optical coherence tomography.

TASS should be considered a potential complication after vitrectomy. This report presents a case of TASS and discusses the differential diagnosis between TASS, infectious and non-infectious endophthalmitis.

## Introduction

Postoperative endophthalmitis (POE) is the most devastating complication of intraocular surgery, and is associated with severe inflammation of ocular issues. POE following vitrectomy is relatively uncommon; in large studies the incidence rates range between 0.02% and 0.15% [1].

Toxic anterior segment syndrome (TASS) is defined as a sterile postoperative inflammation of the anterior segment after intraocular surgery [2]. TASS is usually reported after cataract surgery, however, single studies on TASS after keratoplasty or posterior segment surgeries have been published [3-5]. In large case series involving over 10,000 cataract surgeries, the incidence of TASS was 0.1-0.22% [6-8]. As TASS resembles the symptoms and signs of early infectious POE, an accurate diagnosis can be challenging. The aim of this study is to present a case of TASS after an uncomplicated vitrectomy.

## Case presentation

**Figure 1:**
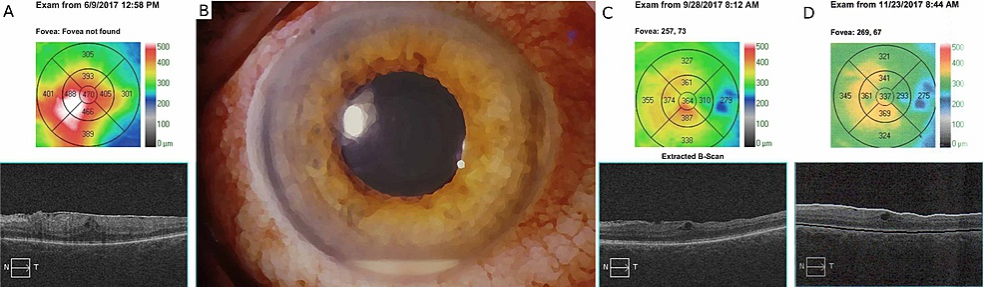
OCT and slit lamp presentation of the reported case (A) Optical coherence tomography (OCT) preoperatively demonstrates a significant epiretinal membrane. (B) An artistic visualization of the case: following vitrectomy, a hypopyon occupying 2 mm of the anterior chamber is present, with no significant corneal edema. Moderate hyperemia can be noted, particularly at the level of pars plana incisions, and a single suture on one of the sclerotomies. OCT scans two (C) and four (D) months after surgery.

A 69-year-old female patient presented to The Elbląg City Hospital, Poland, with an epiretinal membrane (ERM) in the left eye (Figure 1A). She had no significant past medical history and her best-corrected visual acuity in the left eye was 20/100 (right eye was 20/20). Two months later, she underwent a 25-gauge pars plana vitrectomy for ERM peeling in her left eye. During the procedure, careful posterior hyaloid detachment and central vitrectomy were performed. The ERM was stained with MembraneBlue-Dual (Dutch Ophthalmic Research Center, The Netherlands) and removed with end-gripping forceps. No complications were reported, and 2 mg of dexamethasone was injected subconjunctivally at the conclusion of surgery. Postoperatively topical tobramycin 0.3% with dexamethasone 0.1% was prescribed four times daily, while tropicamide 1% and diclofenac sodium 0.1% three times daily. On postoperative day 1 (POD1), the visual acuity had significantly improved to 20/40 and she was discharged from the hospital.

Approximately 30 hours after the procedure, on the evening of POD1, she complained of increasing redness of the eye and vision loss. After a phone consultation, she was recommended to continue her topical treatment, and a visit was scheduled as soon as possible. During the examination on POD3, the visual acuity of her left eye was checked by hand motion. Moderate hyperemia was noted and hypopyon had formed occupying about 2 mm of the anterior chamber (Figure 1B). Despite poor pupil dilation snowbanks in the anterior vitreous and a red reflex from the eye fundus were noted. At this point, due to an early postoperative presentation, no pain or ocular discharge, the diagnosis of TASS was made. Dexamethasone disodium phosphate 2 mg was injected subconjunctivally, and topical dexamethasone 0.1% was recommended six times daily. As it was not possible to exclude POE, on POD4, amoxicillin (875 mg) with clavulanic acid (125 mg) was additionally administered orally and continued for seven days.

Seven days after surgery the symptoms partially resolved; a minimal hypopyon with corneal endothelial deposits was visible, with some snowbanks present in the vitreous cavity. On POD7, 10 and 17, the patient received additional subconjunctival dexamethasone disodium phosphate 2 mg. Subsequently, no symptoms of anterior chamber inflammation were observed on POD17, while mild vitritis was noticed temporally in the anterior vitreous. On POD30, the symptoms of inflammation resolved completely, and the treatment was ceased. Two months after the surgery, slight cystoid changes were noticed in the macula by optical coherence tomography (Figure 1C). Topical nepafenac 0.1% was administered three times daily, resulting in a subsequent decrease of foveal thickness over time (Figure 1D). Visual acuity improved to 20/32; slight opacities within the lens nucleus were observed. Cataract surgery was suggested and the patient was lost to further follow-up.

## Discussion

There is a single case series reporting TASS after vitrectomy with silicone oil administration [5]. In that study, the outbreak of TASS was presumably associated with impurities of the silicone oil batch, and stopped after the batch was changed. In our case, besides anterior segment inflammation, for the first time concurrent vitreous involvement was observed; this is not surprising, as for an ERM peeling a total vitrectomy is not required. 

In the presented case, the diagnosis of TASS and POE should be taken into account (Table 1). One could argue that in the presented case the symptoms resolved with oral antibiotic treatment. The Early Vitrectomy Study (EVS) showed that intravenous antibiotics do not influence the final visual acuity or media clarity in infectious POE [9]. Moreover, in this case, the concentration of antibiotics achieved in the vitreous after oral administration is even lower than with intravenous treatment. POE is a commonly devastating complication of surgery and the visual outcome after in POE is significantly worse than in any kind of sterile inflammatory reaction [4]; in all cases of the EVS, despite an intravitreal injection of amikacin 0.4 mg in 0.1 ml and vancomycin hydrochloride 1.0 mg in 0.1 ml, only 34.2% patients achieved a final visual acuity of 20/40 [9].

**Table 1:** A comparison of clinical manifestation and the final outcome of endophthalmitis and TASS (treated with IV antibiotics). *In cases with visual acuity higher than light perception at presentation, and after intravitreal injection of amikacin 0.4 mg in 0.1 ml and vancomycin hydrochloride (1.0 mg in 0.1 ml). Table created based on findings from [2,9]. Abbreviations: EVS - Endophthalmitis Vitrectomy Study, IV - Intravenous,  TASS - toxic anterior segment syndrome

Clinical manifestation/percentage of cases	Infectious endophthalmitis [9]	TASS [2]	Presented case
Average time from surgery to presentation	6 (1–63) days	Usually within 24-48 hours after surgery	30 hours after surgery
Pain	74.6%	0%–9.5%	No
Perfect media clarity at three-month follow-up	39.3%	Yes, in most cases	Yes
Injection and/or chemosis	82.1%	39.8%–41.4%	Yes
Vitreous body	Progressive vitritis	Not involved	Snowbanks, mild engagement
Hypopyon	81.0%–88.2%	10.6%–100.0%	Yes
Chance of achieving 20/40 acuity without vitrectomy	34.2%*	Yes, in most cases	Achieved
Effectiveness of IV antibiotics	Does not influence outcome	Not required	Administered

Non-infectious endophthalmitis is usually reported after intravitreal triamcinolone acetonide injection; inflammation seen in these cases is believed to be secondary to a reaction to the drug itself, which can be caused by the drug delivery vehicle or a contaminant [10]. Non-infectious endophthalmitis was also reported after intravitreal injections of anti-vascular endothelial growth factor agents or intravitreal steroids. A single report of severe inflammatory response after vitrectomy has been published and was associated with the injection of heavy silicone oil [11]. One should mention this patient did not have a history of uveitis or any other general comorbidities, and the certain reason for the inflammatory reaction in the presented case remains unknown.

TASS development is associated with any substance introduced in the eye during surgery: balanced salt solution, adrenalin, viscoelastics, anesthetics or antibiotics. In our case lactated Ringer’s solution was used for irrigation, just as with every other surgical procedure performed that day and no adverse events were noted in other vitrectomies. Another agent introduced into the eye for ERM staining during vitrectomy was Membrane Blue-Dual (trypan blue 0.15%, brilliant blue G 0.025% on a 4% PEG 3350 carrier). There have been reports of TASS after uneventful cataract surgery using 0.06%-0.1% trypan blue in the anterior chamber for lens capsule staining [12]. However, most commonly TASS is a result of incorrect surgical instrument cleaning or sterilization, including the use of glutaraldehyde, impurities in autoclave steam moisture or personnel changes [13]. The 25-gauge end-gripping forceps were the reusable surgical instrument introduced into the eye in this case; they underwent sterilization within a typical manner in the hospital’s central sterile services department, with no evidence of an abnormal procedure or visible contamination.

The main limitation of this study is that aqueous/vitreous specimen was not obtained and cultured; thus, it is not possible to permanently exclude infectious etiology in this case. On the other hand, despite evident clinical characteristics of infectious POE up to 30.8% of vitreous cultures are negative microbiological tests [9]. With that, infectious endophthalmitis is associated with a poor outcome and even intravitreal antibiotic therapy does not influence the final outcome of the therapy.

## Conclusions

TASS should be considered a potential complication after vitrectomy. This report presents a case of TASS and discusses the differential diagnosis between TASS, infectious and non-infectious endophthalmitis. For an ERM peeling, a total vitrectomy is not required. In this patient, aside from anterior segment inflammation, involvement of the residual anterior vitreous was observed.
